# Supplementary material for: Wnt/β-Catenin Pathway Is Involved in Cadmium-Induced Inhibition of Osteoblast Differentiation of Bone Marrow Mesenchymal Stem Cells
Source: Int J Mol Sci. 2019 Mar 26;20(6):1519. doi: 10.3390/ijms20061519 (PMC6471709; doi:10.3390/ijms20061519)
Supplement: Supplementary file 1 [file ijms-20-01519-s001.zip › Supplementary Files/Table S1.docx]

| **Table S1.** The detailed information of top regulated mRNAs in BMMSCs with CdCl_2_. | | | |
| --- | --- | --- | --- |
| **GeneSymbol** | **Foldchange (CdCl_2_-BMMSCs vs Control)** | **GeneSymbol** | **Foldchange (CdCl_2_-BMMSCs vs Control)** |
| FLOT2 | 0.011285308 | EGR4 | 63.84646436 |
| AMOTL1 | 0.062150871 | TMC5 | 34.06297955 |
| SERPING1 | 0.066315585 | CD24 | 20.70068501 |
| OR11H2 | 0.066745933 | NEFM | 17.77137314 |
| PILRA | 0.0830382 | MT1H | 16.76759339 |
| CTSG | 0.083318507 | BTC | 14.62180817 |
| CBLC | 0.085772133 | ISG15 | 13.54584445 |
| SOAT1 | 0.08758299 | NSUN6 | 12.19905713 |
| CILP | 0.088429355 | DDX24 | 11.18569766 |
| HIF3A | 0.100522245 | MAGEB16 | 10.62420983 |
| SLC35B3 | 0.100534982 | SPINK14 | 9.720137464 |
| BABAM1 | 0.105507758 | VGF | 9.221731079 |
| CEP162 | 0.10951092 | CD320 | 9.029194169 |
| ABI2 | 0.111077479 | PSMB4 | 8.393092233 |
| MAFB | 0.120383831 | LOC730159 | 7.77108374 |
| COX6C | 0.120541477 | CHL1 | 7.637886154 |
| SACM1L | 0.120760601 | CTNNA2 | 7.591055588 |
| TOMM22 | 0.126627458 | NXPE1 | 6.948582164 |
| HOPX | 0.127067446 | CACNA1E | 6.67206691 |
| PNPLA4 | 0.127799196 | KRTCAP3 | 6.640364876 |
| BTRC | 0.128949722 | C1orf146 | 6.636519279 |
| SERPINB9 | 0.1330011 | C12orf60 | 6.554105777 |
| SNX25 | 0.133784614 | ENOPH1 | 6.356288804 |
| ALG5 | 0.13722106 | SEMG1 | 6.281773849 |
| TMA7 | 0.139925795 | ASXL3 | 5.951138297 |
| PPP3CB | 0.152605762 | CANX | 5.925266976 |
| ZNF71 | 0.154553435 | LRRC43 | 5.834440544 |
| TAS2R1 | 0.160379261 | GYPC | 5.726405593 |
| ARHGAP5 | 0.16535833 | TGS1 | 5.72071821 |
| TMEM246 | 0.166355446 | STEAP1 | 5.702442186 |
| INO80B | 0.169287566 | HIST1H2BA | 5.628932204 |
| SLC39A11 | 0.171146339 | SLC2A4RG | 5.613267355 |
| TMEM120B | 0.179947239 | ABTB2 | 5.587245616 |
| MTTP | 0.188783416 | SFT2D2 | 5.484072811 |
| SYNGR1 | 0.190651453 | ZSCAN21 | 5.425400644 |
| UCP2 | 0.209665859 | ZNF536 | 5.395002151 |
| ZFP92 | 0.211325139 | C1QTNF4 | 5.377053323 |
| MAP10 | 0.217092361 | LSM11 | 5.308199583 |
| FBXO15 | 0.222050064 | SOX8 | 5.148449456 |
| RAPGEF5 | 0.222303522 | CASQ1 | 5.056314172 |
