# Supplementary material for: Wnt/β-Catenin Pathway Is Involved in Cadmium-Induced Inhibition of Osteoblast Differentiation of Bone Marrow Mesenchymal Stem Cells
Source: Int J Mol Sci. 2019 Mar 26;20(6):1519. doi: 10.3390/ijms20061519 (PMC6471709; doi:10.3390/ijms20061519)
Supplement: Supplementary file 1 [file ijms-20-01519-s001.zip › Supplementary Files/Table S2.docx]

| **Table S2.** The detailed information of Wnt signaling related mRNAs in BMMSCs with CdCl_2_. | |
| --- | --- |
| **GeneSymbol** | **Foldchange (CdCl_2_-BMMSCs vs Control)** |
| Wnt3a | 0.45765234 |
| β-catenin | 0.331673349 |
| LEF1 | 0.42330398 |
| TCF1 | 0.47767352 |
| FZD4 | 0.327741506 |
| RSPO3 | 0.400303754 |
| AMOTL1 | 0.062150870 |
| RGS20 | 2.965230088 |
| ARL6 | 0.490665339 |
| RTRC | 0.128949721 |
| NOG | 0.441767787 |
| RAPGEF5 | 0.222303522 |
