# Supplementary figures and images for: Wnt/β-Catenin Pathway Is Involved in Cadmium-Induced Inhibition of Osteoblast Differentiation of Bone Marrow Mesenchymal Stem Cells
Source: Int J Mol Sci. 2019 Mar 26;20(6):1519. doi: 10.3390/ijms20061519 (PMC6471709; doi:10.3390/ijms20061519)

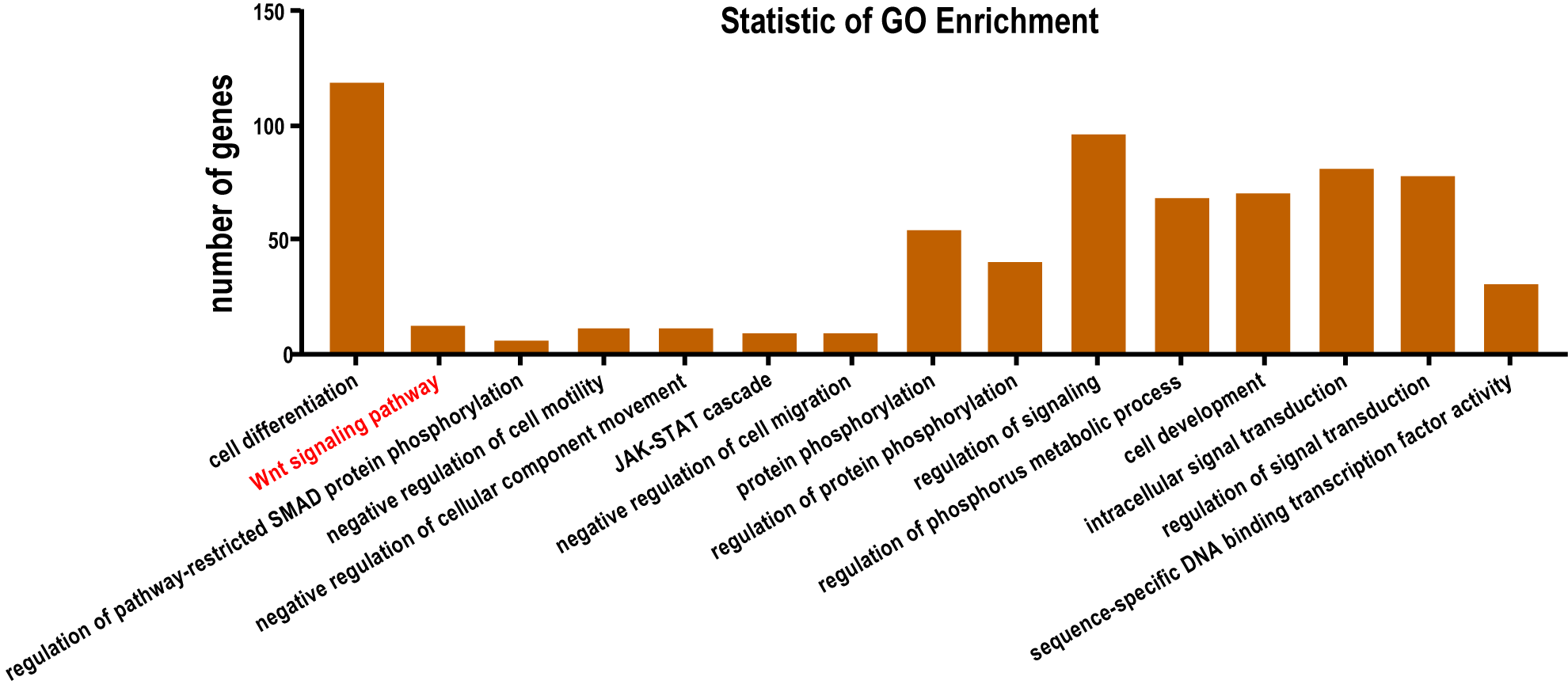

Supplement: Supplementary file 1 [file ijms-20-01519-s001.zip › Supplementary Files/Figure S1.tif]
